# 

**Published:** 1863-12

**Authors:** 


### CONTENTS.

### ORIGINAL CONTRIBUTIONS.

ART. XXXIII. Delayed Union of Fractures, with Cases and Illustrations; the successful employment of Malgaigne's Spike in connection with Drilling, in a case which had previously resisted Drilling employed by itself. By David Prince, M.D., of Jacksonville, Ill., ..... 609
ART. XXXIV. Cases of Cyanosis, attended with, and probably dependent upon, Collapse of the Lung. By John Bartlett, M.D., of Chicago, Ill., ..... 624
ART. XXXV. The Therapeutic Properties of Bromide of Ammonium. By Ira Hatch, M.D., of Chicago, ..... 629
ART. XXXVI. Improved Methods of Treatment in Deformities. By E. Andrews, A.M., M.D., Professor of Surgery in Chicago Medical College, ..... 634

### SELECTIONS.

Employment of Position in Controlling Hæmorrhage, ..... 641

### BOOK NOTICES.

A Practical Treatise on the most Obvious Diseases Peculiar to Horses, By George H. Dadd, V.S., ..... 643

The Principles and Practice of Ophthalmic Medicine and Surgery. By T. Wharton Jones, F.R.S., Prof., &c. ..... 644

Synopsis of the Course of Lectures on Materia Medica and Pharmacy. By Joseph Carson, M.D., ..... 644

A Report on Hospital Gangrene, Erysipelas, and Pyæma. By M. Goldsmith, Surgeon U.S.V., ..... 644

### EDITORIAL.

Clinical Cases in the Medical Wards of the Mercy Hospital, ..... 645
Ice as a Therapeutic Agent in Affections of the Throat. By M. K. Taylor, Surgeon U.S.V. ..... 651
Gleanings from the French Journals, ..... 653
Influence of Steam on Lead, ..... 653
Arsenic in Sulphuret of Antimony, ..... 653
Iodide of Iron and Quinia, Crystalized, ..... 654
Falsification of Essence of Mace, ..... 654
Syrup of Balsam Copaiba, ..... 654
Casein Cement, ..... 655
Sulphate of Protoxide of Maganese, ..... 655
Chlorate of Soda, ..... 655
Hypermanganate of Potossa, ..... 656
Royal Humane Society's Instructions, ..... 657
Drowning, Instructions for Restoring Persons, ..... 657
Investigations touching the Use of Iodine, ..... 659
Inquests in England for 1862, ..... 660
Sclerotitis and Iritis, ..... 659
Health of San Francisco, ..... 661

